# Effects of a Multicomponent Exercise Program in Older Adults with Non-Small-Cell Lung Cancer during Adjuvant/Palliative Treatment: An Intervention Study

**DOI:** 10.3390/jcm9030862

**Published:** 2020-03-21

**Authors:** Ilem D. Rosero, Robinson Ramírez-Vélez, Nicolas Martínez-Velilla, Bernardo Abel Cedeño-Veloz, Idoia Morilla, Mikel Izquierdo

**Affiliations:** 1 Department of Health Sciences, Public University of Navarra, Navarrabiomed-Biomedical Research Centre, IDISNA-Navarra’s Health Research Institute. C/irunlarrea 3, Complejo Hospitalario de Navarra, 31008 Pamplona, Navarra 31008, Spain; ilemdayana@gmail.com (I.D.R.); robin640@hotmail.com (R.R.-V.); nicolas.martinez.velilla@navarra.es (N.M.-V.); ba.cedeno.veloz@navarra.es (B.A.C.-V.); idoiamorilla@gmail.com (I.M.); 2 Biomedical Research Networking Centre on Frailty and Healthy Ageing, CIBERFES, 28029 Madrid, Spain

**Keywords:** exercise therapy, physical function, strength, functional capacity, lung cancer, elderly

## Abstract

Clinical intervention studies support the efficacy and safety of exercise programs as a treatment modality for non-small-cell lung cancer (NSCLC) during adjuvant/palliative treatment, but the effectiveness of real-world oncogeriatric services is yet to be established. We aimed to examine the effects of a 10-week structured and individualized multicomponent exercise program on physical/cognitive functioning and mental wellness in elderly patients with NSCLC under adjuvant therapy or palliative treatment. A non-randomized, opportunistic control, longitudinal-design trial was conducted on 26 patients with NSCLC stage I–IV. Of 34 eligible participants, 21 were allocated into two groups: (i) control group (*n* = 7) received usual medical care; and (ii) intervention group (*n* =19) received multicomponent program sessions, including endurance, strength, balance, coordination and stretching exercises. Tests included the Short Physical Performance Battery (SPPB), 5-m habitual Gait Velocity Test (GVT), Timed Up & Go Test (TUG), 6-Min Walk Test (6MWT), independence of activities in daily living (IADL), muscular performance, cognitive function, and quality of life, which were measured at baseline and after 10 weeks of the program. Results revealed a significant group×time interaction for SPPB (*p* = 0.004), 5-m GVT (*p* = 0.036), TUG (*p* = 0.007), and muscular performance (chest and leg power; *p* < 0.001). Similarly, significant changes were observed between groups for cognitive functioning (*p* = 0.021) and quality of life for EUROQoL 5D (*p* = 0.006). Our findings confirm that a multicomponent exercise program improves measures of physical/cognitive functioning and quality of life in the elderly with NSCLC under adjuvant therapy or palliative treatment. This is an interesting and important study that adds to our current body of knowledge on the safety of exercise interventions, especially in the elderly with solid tumors.

## 1. Introduction

Lung cancer remains a leading cause of mortality worldwide among men and women, with 2.1 million new lung cancer cases and 1.8 million deaths predicted in 2018, representing almost 1 in 5 cancer deaths (18.4%) [1]. Lung cancer is the second most prevalent type of cancer and is broadly divided into two categories based on histological characteristics: small cell lung cancer (SCLC, representing 20–25% of all diagnosed lung cancer cases) and non-small-cell lung cancer (NSCLC, 70–85% of cases) [2]. The most important risk factor for lung cancer is tobacco smoking, although other causes include environmental pollutants [3]. The probability of developing lung cancer increases considerably as patients age, accounting for ~60% of all new cases according to the International Agency for Research on Cancer GLOBOCAN 2012 project [1]. The majority of patients with lung cancer (~75%) have incurable locally advanced or metastatic cancers at the time of diagnosis and have a mean five-year mortality rate of 85–90% [4].

Cancer is primarily a disease of the elderly. Elderly patients with NSCLC experience a complex set of symptoms such as dyspnea, fatigue, shortness of breath, weight loss and pain, as well as distress caused by anticancer treatment, the disease itself, and psychological distress in the form of resignation, anxiety, and/or depression [5]. In developed countries, people aged 75 and over already represent around one third of cancer patients, and incidence rates are increasing with age for most tumors [4,5,6]. All of these features have a likely effect on physical function/performance and will have a negative impact on health-related quality of life (HRQoL), functional status, and the ability to participate in activities of daily living (ADLs) [6]. Thus, improving psychosocial well-being to enhance HRQoL and physical function is a primary goal at all stages of lung cancer during treatment and survivorship [7].

Adjuvant therapy (additional cancer treatment) may include chemotherapy, radiation therapy, hormone therapy, targeted therapy, or biological therapy. In addition, the therapeutic options for advanced NSCLC are based on numerous factors including histological characteristics, stage of the disease, and the patient’s performance status [8], and can involve different strategies such as: (i) minimal invasive surgery by video-assisted thoracoscopic surgery (VATS); (ii) chemotherapy, chemoradiotherapy, immunotherapy (neoadjuvant), targeted therapy, either in combination or in isolation; (iii) palliative care; (iv) comprehensive rehabilitation program; (v) physical exercise [9]. However, these options are limited in many cases because of poor functional status including older age, comorbidities, sedentary behavior, poor exercise capacity, and loss of muscle strength.

Strategies that complement advances in conventional cancer treatment, especially those that reduce treatment-related morbidities, are of major clinical importance in the oncogeriatric population. This could be because reduced functional ability in many elderly is a result of critical deterioration of a complexity of function, for example, balance, endurance, muscle strength, coordination, and reaction capacity [7,9]. It is therefore likely that a supervised exercise program with the aim of improving these functions would be required in order to improve the ability to perform basic daily tasks in general. However, many studies have focused on the impact of intensive single-component training on isolated functions in laboratory settings [10,11,12].

Recent years have witnessed a growing interest in non-invasive interventions for patients with lung cancer, with the goal of maximizing exercise performance capacity and other outcomes such as muscle strength and HRQoL, and decreasing emotional distress [10]. Recently, multicomponent exercise programs have been demonstrated to be safe and well tolerated, but there remains a paucity of data to draw conclusive and precise exercise guidelines [11,12]. For example, a recent Cochrane review of six randomized controlled trials (*n* = 221) failed to establish any conclusive evidence regarding efficiency of exercise training on physical fitness and other outcomes such as cardiorespiratory capacity, muscle strength, mental wellness, and HRQoL in patients with advanced lung cancer [12]. Thus, the effectiveness of multicomponent exercise programs on these outcomes in these patients is unclear [13]. Likewise, the effectiveness of exercise training in improving other outcomes, such as physical functioning, cognitive functioning, and mental wellness is yet to be demonstrated.

Therefore, the objective of this research was to examine the effects of a 10-week structured and individualized multicomponent exercise program on physical/cognitive functioning and mental wellness in elderly patients with NSCLC under adjuvant therapy or palliative treatment. Accordingly, the research question for this intervention study was: does a structured and individualized multicomponent exercise program improve physical/cognitive functioning and HRQoL outcomes more than usual care among the elderly with NSCLC?

## 2. Methods

### 2.1. Study Design, Setting and Ethical Considerations

This was a non-randomized, opportunistic control, longitudinal trial designed to examine the effects of a multicomponent exercise program on surrogate measures of health status in patients with lung cancer in real-world settings. Patients were treated at the Oncogeriatrics Unit of the Complejo Hospitalario de Navarra (CHN). The study ran from May 2018 to November 2019 and was approved by the CHN Research Ethics Committee (25, April, 2018, reference number Pyto2018/5#214) according to the principles of the World Medical Association Declaration of Helsinki. Verbal explanations of the aims and intervention of the study were provided to the patients, and those who were willing to participate signed a written informed consent form.

### 2.2. Patient Population

We enrolled newly diagnosed patients with NSCLC stage I–IV (Tumour, node, metastasis (TNM) classification) [14], histologically confirmed at CHN hospital and approached consecutively, advised about the study, screened for eligibility (if willing) and invited to participate in the study (if eligible) by physicians for curative or palliative purposes. The study included an initial exam at the first visit (baseline) and a final exam after 10-weeks. A trained research assistant conducted a screening interview to determine whether potentially eligible patients met the following inclusion criteria: aged 70 years or older; have a diagnosis of confirmed lung cancer; with life expectancy exceeding three months (prognosis); with multimorbidity, presence of geriatric syndromes or fragility (VES13/ G8 Index); Barthel score ≥60 points; and able to communicate and collaborate with the research team. Exclusion criteria were: clinically unstable patients defined medically as having received active treatment (chemotherapy or radiotherapy) for the neoplasm before inclusion in the study; moderate-severe cognitive impairment considered as a score ≥5 in the Reisberg Global Deterioration Scale; and contraindications to exercise, or already engaged in high levels of physical training. All testing procedures were carried out at the biomedical research center of the Government of Navarre (Navarrabiomed) and CHN, Universidad Pública de Navarra, Pamplona, Spain. Participants received no monetary incentive. The trial protocol was developed according to the Standardized Protocol Items: Guidelines for reporting non-randomized studies [15]. Due to the nature of the intervention, participants and researchers were not blinded; however, the principal investigator was not involved in the exercise training and analyses were performed blinded for group allocation. Each participant was assessed in the same period of the day (morning or afternoon) in a metabolic and sport science research laboratory by the same assessor.

### 2.3. Outcome Assessment

After enrollment, patients were invited to visit Navarrabiomed Lab to collect baseline data. The baseline data included anthropometry (weight and height), prognosis, health status, polypharmacy, geriatric syndromes or fragility status, cognitive impairment, clinically diagnosed comorbidities, tumor node metastasis classification, adjuvant therapy/palliative treatment, and dependence. Height was measured to the nearest 0.1 cm using a stadiometer and body weight to the nearest 0.1 kg using digital scales. Body mass index (BMI) was also calculated.

The primary outcome was objectively measured physical functioning using the “Short Physical Performance Battery” (SPPB) [16]. To assess usual walking speed (meters/second), the participants were asked to walk 4 m at their regular pace twice from a standing position. The standing balance tests included side-by-side, semi-tandem, and full-tandem standing, and the participants were timed until they moved, or 10 s had elapsed. To assess the 5-times sit-to-stand test, the participants were asked to perform five chair stands as quickly as possible. Time (in seconds) was registered with a stopwatch with a resolution of 0.01 s. The total score ranged from 0 (worst) to 12 points (best). An increase of 1-point is recommended in disability research [16]. In this context, quantifying physical performance status after cancer treatment may provide important insights to clinicians on the health, vitality, and prognosis of cancer survivors [17].

The secondary outcomes were physical functioning by: (i) 5-m habitual gait velocity test (GVT); (ii) timed up and down stairs test (TUDs); (iii) 6-min walk test (6MWT); (iv) dependence; (v) muscular performance; (vi) cognitive functioning; (vii) mental wellness, which are commonly used measurements of functional/exercise capacity in patients with pulmonary morbidity or cancer [13]. For the GVT, participants were instructed to walk at their usual pace. All clinical outcome measures (minimum clinical difference or validity test) included in this study has been previously reported in similar population and real-world settings [13,17,18,19,20,21,22,23,24,25,26,27,28,29,30,31,32]. Dual-task conditions (gait evaluation during the simultaneous performance of a cognitive task) have recently been recognized as a sensitive assessment method for interactions among cognition, gait, falls, and frailty [18]. Two trials were conducted to assess gait velocity while the patient performed a verbal or counting task (verbal GVT and arithmetic GVT, respectively). During the verbal dual-task condition (verbal GVT), the gait speed was measured while the participants named animals aloud. During the arithmetic dual-task condition (arithmetic GVT), the gait speed was evaluated while the participants counted backwards aloud from 100 in ones.

After the familiarization sessions, the following tests validated for this population were performed: TUG: rom a starting position with the hips, knees, and ankles flexed at 90°, the participants had to rise from a chair, with no armrests, walk 3 m, return, and sit back in the chair. Participants were instructed not to run. The 6MWT was used as a measure of physical function in older adults [19]. Briefly, patients were instructed to walk as far as possible along a short-measured course during 6 min in a 30-m field [20]. The time in all of the tests was measured by the same evaluator and with the same chronometer to the nearest 0.1s. Dependence was assessed with an ADLs evaluation using a Spanish-adapted version of the physical level activities of daily living (Barthel score), recommended for epidemiological studies in the elderly [21]. The items of the Barthel Index are weighted: a maximum score of 100 indicates independence, 91–99 minimal dependence, 75–90 mild dependence, 50–74 moderate dependence, 25–49 severe dependence, and 0–24 total dependence [22].

Used widely in studies of patients with cancer, muscle strength was measured by the maximal amount of weight that each muscle group (lower extremity (leg press) and upper extremity (chest press) can move through the available range of motion (1 repetition maximum (1RM)) using eGym^®^ equipment (eGym^®^ GmbH, München, Germany)). Maximal strength was assessed by a handgrip test (Jamar, Sammons Preston Rolyan, Nottinghamshire, UK), whereas knee extension, flexion, and abduction hip were determined using a digital dynamometer (Hoggan Scientific LLC, Salt Lake City, UT; Micro FET 2 muscle testing). Two trials were performed and the average was used for subsequent analyses.

Cognitive functioning was assessed by several tests. The mini-mental state examination (MMSE) assesses domains of orientation, memory, attention, language, and visuospatial ability, with a maximum score of 30 points [23]. Scores ≤23 points are indicative of likely cognitive impairment. In the verbal fluency test, the patient had 1 min to enumerate as many words as possible starting with the letter F [24]. The Trail Making Test, part A (TMT-A) is an indicator of visual scanning, graphomotor speed, and executive function. In this case, patients were asked to connect randomly arranged circles containing numbers from 1 to 25 following the number sequence as quickly as possible [25].

Self-perceived physical function and health status were evaluated using the HRQoL and questions on cancer-related symptoms. These questionnaires included the European Quality of Life-5 Dimensions (EUROQoL 5D) and the “European Organization for Research and Treatment of Cancer Quality of Life Questionnaire” (EORTC QLQ) [26]. This last instrument combined the general part (QLQ-C30) and the lung-specific part (QLQ-LC13). The reliability and validity of the EORTC QLQ-C30/LC13 has been reported for exclusive use in patients with lung cancer [27]. It is composed of both multi-item scales and single-item measures that contain five independently functional subscales (physical, role, cognitive, emotional, and social); three symptom subscales (fatigue, pain, and nausea and vomiting); and a global health/QoL subscale. Furthermore, it comprises six single items assessing symptoms commonly reported by cancer patients (dyspnea, appetite loss, sleep disturbance, constipation, and diarrhea). This test establishes an intuitive association between categories and a numerical equivalent, such as 0-Nothing, 4-Little, 6-Some, 10-Much [28]. A short version of the Geriatric Depression Scale (GDS) [29] developed in 1986, was used, and included 15 relevant questions selected from a longer GDS version regarding the highest correlation with depressive symptoms in validation studies. Scores of 0–4 are considered normal, according to age, education, and complaints; 5–8 indicates mild depression; 9–11 indicates moderate depression; and 12–15 indicates severe depression [30]. Finally, Borg’s perceived exertion scale was used, which measures the extent of perceived exertion that a person experiences during exercise [31]. Physical and mental outcomes were tested by a highly qualified Master of science-level physiotherapist with 15 years of experience.

Members of the research team were able to access the medical records of each patient, which contained important data such as tumor node metastasis classification, frailty status (G8 and VES 13), alcohol and smoking status (current, past or no), surgery type (VATS, open, without surgery), and adjuvant therapy/palliative treatment (chemotherapy, immunotherapy, radiotherapy, immunotherapy and palliative treatment). The same assessments were repeated at 8- and 10-weeks after intervention or usual care. All outcomes were collected at Navarrabiomed via face-to-face interviews by trained health workers.

### 2.4. Intervention

Usual nursing and medical care were provided to all participants in all groups. The control group (CG) included participants who were not referred to the Oncogeriatrics services or who were on an extended waiting list for oncology treatment and were willing to undergo an assessment of physical function and clinical status at baseline and after 10 weeks. These participants had not practiced any kind of supervised physical exercises/activities during the intervention 10-weeks, but received normal outpatient care, including physical rehabilitation when needed.

The intervention group (IG) receiving the multicomponent exercise program was carried out in the Navarrabiomed Lab facilities. In this line, the most beneficial type of physical exercise is called multicomponent exercise, according to feasibility and safe intervention from our lung cancer study. [13,18,32]. It is recommended that the multicomponent exercise program is individualized according to treatment of the cancer patient and their general health condition. This type of program combines strength (resistance), endurance (aerobic), balance/coordination, and flexibility training and has been shown to result in great improvements in functional capacity, which is a key point in maintaining independence in instrumental and basic activities of daily living. Each session lasted 45–50 min and the exercise protocol was performed twice a week over 10 weeks. Attendance was registered by the patients at every exercise session in an exercise diary logbook kept at the Navarrabiomed Lab Centre. Adherence to the exercise intensity was monitored by HR monitor (Polar Team, A2 Polar Electro Oy, Kempele, Finland). A highly qualified physiotherapist (I.D.-R.) supervised each training session. The exercise program was individualized and included measurements of vital signs at the beginning and end of each session (oxygen saturation-SpO_2_, heart rate-HR, blood pressure-BP). The program contained endurance exercise, such as classic recumbent exercise bike (R20 Vision Fitness Comfort Arc™, Taichung, Taiwan,) 50–80% of HR, squeezing a ball (3–5 min); getting up from a chair (3 sets/10 repetitions); balance and coordination (2 sets); eGym^®^ machines (Model M5 and M9 eGym equipment ^®^ Berlín Germany), maximum strength in chest press (1RM), power chest (3 sets/12 repetitions at 30% of 1RM), maximum strength in leg press (1RM), and power legs (3 sets/12 repetitions at 30% of 1RM); hip abduction with elastic bands (3 sets/10 repetitions); and general stretching exercises (5 min). The amount of chest and leg press ranged from 30% to 60% of 1RM and 8–12 repetitions according to the training week. The external load was adjusted weekly to maintain the % of the 1RM (from 30% to 60% of 1RM) and total number of repetitions per exercise (8 to 12 repetitions). The intensity of the exercises increased individually and progressively according to the patient’s response, pain, dyspnea, and feelings of well-being or fatigue on each day of exercise. Additionally, assistance was provided to subjects during the exercise to complete the proposed RM. Patients were advised to carry out the “Vivifrail” [32] program at home during the entire study period. The program is personalized, depending on the older person’s functional capacity level (serious limitation, moderate limitation, and slight limitation as evaluated by the SPPB, and a walking speed test) and the risk of falling [32]. This type of intervention has also been proven as the most effective to delay disability, cognitive impairment, and depression as well as effective in reversing the functional decline associated with acute hospitalization in very old patients, (Table 1) [32]. The multicomponent exercise protocol and “Vivifrail” [32] program comply with the American College of Sports Medicine exercise guidelines for cancer in relation to improving physical fitness and restoring physical functioning, enhancing quality of life, and mitigating cancer-related fatigue [18].

### 2.5. Statistical Analyses

All analyses were performed by a researcher who was not involved in the study participants’ assessments and interventions. The statistical data analysis was performed with the commercial software SPSS Statistics version 25.0 (IBM Corp., Chicago, IL). The Shapiro-Wilk test was used to determine whether parametric tests were appropriate. Normality of data was checked graphically. In the present study, descriptive data, including frequencies for categorical variables and means standard deviation (SD) for continuous variables, were reported. Baseline differences were analysed using chi squared (X^2^) test for nominal data and the Kruskal–Wallis test for ordinal data. The change value was calculated post-pre (∆) with 95% confidence intervals (95%CI), to compare differences between the groups. Non-parametrical data were analyzed with the Wilcoxon and Mann-Whitney U tests. For parametric data, Student’s t tests were used. A significance level of 5% (*p* < 0.05) was adopted for all statistical analyses.

## 3. Results

### 3.1. Characteristics of Participants

**Figure 1:**
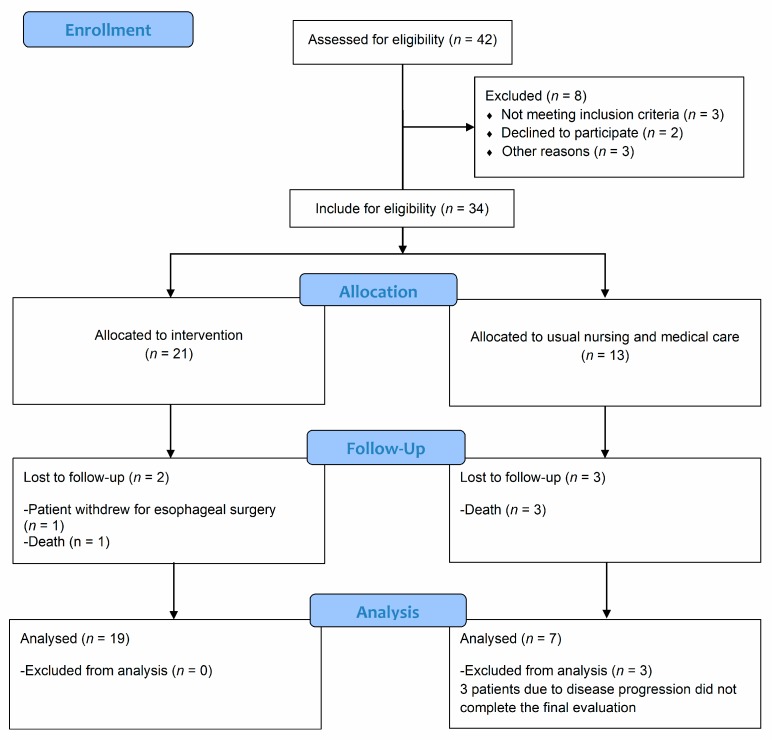
CONSORT Flow Diagram - modified for non-randomized trial design.

Of the 42 volunteers, 34 attended the oncologic and geriatric clinics screening. Of these, 26 completed the 10-weeks intervention. Two patients from the IG did not complete the program due to death or esophageal surgery. Data from the 19 remaining patients from the IG were analyzed. A total of 6 of the 13 CG subjects dropped out of the study and did not take the final exam due to the progression of the disease (three) or death (three). Data from the seven remaining CG participants were analyzed. A total of 19 participants (4 females, 15 males) were eligible for analysis in the IG and 7 participants (2 females, 5 males) in the CG (Figure 1). All subjects in the IG completed at least 86% of the planned training sessions. No major adverse events or health-related issues attributable to the testing or training sessions were noted.

Table 2 displays the baseline characteristics by group. No significant differences were found between the two groups, except for age. Patients in the IG had a mean (SD) age of 74.5 (3.6) years, in the range 70–81 years (78.9% males), and BMI 26.8 (4.5) kg/m^2^. In total, 41.2% underwent surgery and 78.9% received adjuvant chemotherapy alone or in combination with other therapies. Participants in the CG had a mean (SD) age of 79.0 (3.0) years, in the range 75–83 years (71.4% males), and BMI 25.5 (2.5) kg/m^2^. Within this group, 14% were submitted to surgery and 85.7% were receiving adjuvant chemotherapy alone or in combination with other therapies.

### 3.2. Intervention Effects

Table 3 shows the results of physical functioning in both groups and the differences between groups after the 10-week program. The IG showed a significant increase in the following functional capacity tests: SPPB gait speed test 4-m (*p* = 0.008), chair stand test (*p* = 0.009), total score (*p* = 0.004), and the 5-m GVT (*p* = 0.036). Additionally, the IG group showed significantly improved muscle performance (strength) measured as leg press (*p* < 0.001), chest press (*p* < 0.001), and hip abduction (*p* = 0.001). By contrast, there was a significant decrease in functional capacity in the CG: 5-m GVT (*p* = 0.020), GVT verbal (*p* = 0.017), TUG (*p* = 0.016). Additionally, there was a significant decrease in muscle performance in 1RM chest press (*p* = 0.049) and chest power (*p* = 0.022). A significant difference was found in functional capacity between groups: SPPB gait speed test 4-m, chair stand test, total score (*p* = 0.002); 5-m GVT (*p* = 0.005), GVT verbal (*p* = 0.006), GVT arithmetic (*p* = 0.012); TUG (*p* = 0.007); and Barthel index (*p* = 0.044). There was a significant difference in muscle performance (strength) for leg press (*p* < 0.001), chest press (*p* = 0.001), and flexion hip (*p* = 0.024).

Table 4 shows the results of cognitive functioning within the two groups and the differences between groups. The significant increase in MMSE observed in the IG (p=0.005) resulted in a significant difference between groups (*p* = 0.021).

Table 5 shows the results of HRQoL domains within and between both groups. The CG did not show major differences, whereas the IG showed a significant improvement in the EUROQoL 5D questionnaire in total score (*p* = 0.038), EORTC QLQ-C30 questionnaire in physical function (*p* = 0.037) and global health status/quality of life (*p* = 0.029), and a significant decrease in pain symptoms (*p* = 0.030) and dyspnea (*p* = 0.025), as well as pain in other body parts (*p* = 0.025) in the EORTC QLQ-LC13 questionnaire. The significant difference between groups was found for EUROQoL 5D questionnaire in total score (*p* = 0.006), and pain in other body parts (*p* = 0.007).

## 4. Discussion

Supervised exercise training can be beneficial for patients with lung cancer by increasing functional capacity, exercise tolerance, and physical performance/fitness, and by reducing emotional distress. Our findings confirm our hypothesis that a multicomponent exercise program in the elderly with NSCLC under adjuvant therapy or palliative treatment positively affects measures of functional capacity measured by SPPB, GVT, and muscular performance (1RM and power). Our results also suggest that such an intervention improves cognitive functioning (MMSE score) and quality of life (physical function, pain symptoms, and dyspnea). We believe that the present study represents an important addition to the current body of knowledge on the safety of exercise interventions, particularly in the elderly with NSCLC under adjuvant therapy or palliative treatment.

The safety, benefits, and application of a multicomponent program in patients with lung cancer with the goal of improving physical and mental outcomes have been previously established [10]. Rosero et al. [13] reported a strong negative relationship between exercise capacity, including sub-maximal oxygen consumption, and several emotional issues (quality of life, depression and fatigue) and medical care (days of hospitalization and postoperative complications) in patients with NSCLC. In our study, we found differences in physical performance between groups (post-pre change 2.53; 95% CI 1.05 to 4.02) measured by the SPPB, despite the aggressive nature of the disease and its treatment. Previous studies have reported that impaired mobility is reflected by total SPPB scores of less than 10, and those patients with total SPPB scores of 7–9 are 1.6 to 1.8 times more likely to become disabled [33,34]. The 6MWT is one of the most common measures in studies on lung cancer and exercise. In the present study, the minimum significant difference in individuals with lung cancer (22–42 m) was not achieved, although there was an increase of 5.4 m on average between initial and final evaluation in the IG, compared with a decrease of 27.2 m in the CG. Similarly, Dhillon et al. [35] reported an increase in 6MWT from baseline to post-intervention in the exercise group (234.9 m to 516.3 m) as well as the control group (251.0 m to 517.7 m), but there was no significant between-group difference (*p* = 0.972).

The improved function and metabolism of skeletal muscle is likely to explain the improvements in functional capacity, including the 4- and 5-m walk tests, getting up from a chair, total SPPB score, and muscle performance. Similarly, skeletal muscle function improves significantly after a multicomponent exercise program in older adults [24]. Loss of skeletal muscle function results in reduced physical performance during sub-maximal exercise as well as a reduced capacity to oxidize fats as a fuel, and this is in part due to a reduction of muscle mitochondria in NSCLC patients.

In clinical studies on patients with lung cancer, several authors have reported cognitive deficits early after following chemotherapy treatment, particularly on executive function, verbal fluency, and verbal memory [36,37]. Whilst chronic exercise seems to improve memory, verbal concept formation, selective attention, and conflict resolution, studies have focused mainly in older adults [35]. We found a significant improvement in cognitive function by MMSE, a key component of the intrinsic capacity of individuals that is a combination of all physical and mental abilities, which helps to preserve autonomy and independence in essential daily activities [38].

Overall, the possible positive effects of chronic physical exercise on executive functions may be explained by the improved structural connectivity of the prefrontal brain areas. It has been shown that white matter integrity in the prefrontal cortex is important for executive functioning [39]. Among the different components of executive function, the present study focused on cognitive inhibition and attention capacity. Mechanisms such as angiogenesis, synaptogenesis, and neurogenesis have been proposed as possible mediating factors in the exercise-cognition relationship. Specifically, several hypotheses may explain this relationship: *i)* regulation of neurotrophins (such as growth factors, brain-derived neurotrophic factor, neurotrophin-3, and neurotrophin-4/5); *ii)* increases in oxygen saturation due to increased blood flow and circulatory angiogenesis; and *iii)* increases in brain neurotransmitters (e.g., norepinephrine and serotonin) facilitating information processing [40]. This is likely to explain the association between exercise training and higher executive function.

Emerging evidence suggests that structured exercise can confer protection against the emergence of mental illness and be used as an adjunctive treatment in several chronic diseases [41]. Moreover, meta-analyses have demonstrated small but significant improvements in quality of life following exercise training in cancer survivors [42,43]. Of note, these reviews of cancer survivors contained few participants with advanced lung cancer [11]. With regard to quality of life, we found a significant improvement in physical functioning, symptom levels (pain and dyspnea), and significant improvements in HRQoL in those patients whose performance status had improved. Preliminary studies suggest that supervised exercise and multicomponent programs may benefit mental health, with significant improvements in reducing the severity of depression and anxiety in cancer survivors [44,45,46,47]. In the present study, significant differences between groups were found only for pain in other body parts (*p* = 0.007). Using the EORTC-QLQ-C30 questionnaire, Dhillon et al. [35] found no difference in HRQoL score (*p* = 0.817) between the supervised exercise group (63.8 to 63.2) and the control group (58.9 to 64.3) on completion of an eight-week intervention. In this respect, further research to determine the effects and optimal dosage of exercise programs in this particular population is warranted.

In this study, differences were observed in the groups according to age. However, although age is an important factor that has a significant impact on the quality of life and mortality in the elderly population with lung cancer, this difference is relatively small, in part due to design of non-randomized design trial and we were encouraged to reinforce our findings.

## 5. Limitations

Exercise is widely recommended to cancer patients. However, knowledge of the optimal parameters of physical exercise (mode, intensity, frequency, duration, and time) is lacking for patients with NSCLC, and so it is difficult for physicians to correctly prescribe physical exercise. Our study uses an exercise protocol that could be followed to obtain benefits on physical, cognitive functioning, and mental well-being. It is also one of the few studies that assesses whether a supervised and individualized physical intervention is beneficial and safe for patients with advanced NSCLC under adjuvant therapy or palliative treatment. None of the previous studies that have evaluated physical training in older adults with lung cancer reported serious adverse events [46,47,48,49,50,51,52], which is consistent with the findings of our study.

This study has several limitations that should be considered before interpreting these findings. Firstly, the number of participants in our study was relatively small, and so larger multicenter studies are encouraged to reinforce our findings. A second limitation was the poor condition of three patients in the CG that prevented the evaluation of the changes from the study beginning to end, which could have revealed significant differences in other measurement variables. While there were no adverse events, the sample may be below the number needed to show harm. These limitations are important and should be addressed in the future.

## 6. Conclusions and Future Recommendations

In conclusion, patients with NSCLC have a clear indication of starting a multicomponent exercise program during adjuvant therapy or palliative treatment for their disease. Our findings point to several future directions for research. In general, the physical exercise intervention seems to provide physical, cognitive, and emotional benefits in older adults with NSCLC under adjuvant therapy or palliative treatment. Future studies should analyze the cellular benefits in this population, and also study the correlation between physical exercise and the toxicity of cancer treatments. Well-designed randomized clinical trials should be performed to corroborate the current findings with a larger sample size to detect a significant difference in the components studied.

## Figures and Tables

**Table 1:** Multi-component exercise program.

*n*	Exercise	Frequency	Duration	Intensity
1.	Endurance exercise: riding a bicycle	Twice a week	3–5 min	50–80% of HR max
2.	Get up from a chair	Twice a week	3 sets × 10 repetitions	−
3.	Balance and coordination Walking on toes with flexion-extension shoulder Walking on heels of the feet with abduction-adduction shoulder Progression with obstacles along the way	Twice a week	2 sets × 5 m	−
4.	Maximum strength/power in chest press (eGym^®^)	Twice a week	2 times (for control load)	1RM
5.	Maximum strength/power in leg press (eGym^®^)	Twice a week	2 times (for control load)	1RM
6.	Power chest (Explosive program eGym^®^)	Twice a week	Week 1 3 sets/12 repetitions	30% RM
			Week 2–3 3 sets/12 repetitions	40% RM
			Week 4–7 3 sets/10 repetitions	50% RM
			Week 8–10 3 sets/8 repetitions	60% RM
7.	Power legs (Explosive program eGym^®^)	Twice a week	Week 1 3 sets/12 repetitions	30% RM
			Week 2–3 3 sets/12 repetitions	40% RM
			Week 4–7 3 sets/10 repetitions	50% RM
			Week 8–10 3 sets/8 repetitions	60% RM
8.	Hip abduction with elastic bands (TheraBand)	Twice a week	3 sets/10 repetitions	50–80% band elongation (red-black)
9.	Squeezing a ball	Twice a week	3 sets/3–5 min	−
10.	General stretching exercises (6–10 exercises)	Twice a week	5 min	−

Abbreviations: HR: Heart Rate; RM: Repetition Maximum.

**Table 2:** Baseline characteristics of the participants.

Variables	Intervention Group (*n* = 19)	Control Group (*n* = 7)	*p*-Value
Age	74.5 (3.6)	79.0 (3.0)	0.007 *
BMI, kg/m^2^	26.8 (4.5)	25.5 (2.5)	0.491 *
Underweight, *n* (%)	5 (26.3)	1 (14.3)	0.912 †
Normal weight, *n* (%)	7 (36.8)	4 (57.1)	
Overweight/obesity, *n* (%)	7 (36.8)	2 (28.6)	
Sex, *n* (%) Male Female	15 (78.9) 4 (21.1)	5 (71.4) 2 (28.6)	0.700 +
TNM classification, *n* (%) I II IIIa-IIIb-IIIc IV	1 (5.3) 0 (0.0) 4 (21.0) 14(73.7)	0 (0.0) 1 (14.3) 3 (42.8) 3 (42.8)	0.172 †
Clinically diagnosed comorbidities, *n* (%)			0.496 +
COPD	13 (68.4)	2 (28.6)	
Hypertension	9 (47.4)	4 (57.1)	
Diabetes mellitus	5 (26.3)	1 (14.3)	
Cardiovascular diseases	8 (42.1)	4 (57.1)	
Frailty status, *n* (%) G8 VES 13 G8 and VES13 No	7 (36.8) 2 (10.5) 4 (21.1) 6 (31.6)	3 (42.8) 0 (0.0) 3 (42.8) 1 (14.3)	0.649 +
Alcohol status, *n* (%) Current Past No	10 (52.6) 4 (21.1) 5 (26.3)	2 (28.6) 3 (42.8) 2 (28.6)	0.407 +
Smoking status, *n* (%) Current Past No	5 (26.3) 11 (57.9) 3 (15.8)	2 (28.6) 3 (42.8) 2 (28.6)	0.763 +
Surgery, *n* (%) VATS Open Without surgery	3 (17.6) 4 (23.5) 12 (58.8)	0 (0.0) 1 (14.3) 6 (85.7)	0.144 +
Adjuvant therapy/palliative treatment, *n* (%) Chemotherapy Immunotherapy Chemotherapy and radiotherapy Chemotherapy and immunotherapy Immunotherapy and radiotherapy Palliative treatment	6 (31.6) 1 (5.3) 7 (36.8) 2 (10.5) 2 (10.5) 1 (5.3)	2 (28.6) 1 (14.3) 4 (57.1) 0 (0.0) 0 (0.0) 0 (0.0)	0.385 +

Abbreviations: BMI, body mass index; COPD, chronic obstructive pulmonary disease; TNM, tumor node metastasis; VATS, video-assisted thoracic surgery; VES-13, Vulnerable Elders Survey-13. Data are reported as mean ± standard deviation or number (%). Differences between groups were analysed using * U Mann Whitney for nominal data, + X^2^ test for nominal data, or † the Kruskal–Wallis test for ordinal data.

**Table 3:** Results of physical functioning outcomes by group.

	Intervention Group (*n* = 19)			Control Group (*n* = 7)			Group Difference ∆ (95% CI) *p*-Value Groups +
	Before	After	*p*-Value *	Before	After	*p*-Value *	
	Mean (SD)	Mean (SD)		Mean (SD)	Mean (SD)		
**Functional capacity**							
Gait Speed Test 4-m (s)	5.62 (1.57)	4.89 (1.19)	0.008	4.85 (0.83)	5.71 (0.87)	0.051	−1.60 (−2.55 to -0.65); 0.002
Static Balance (Score)	3.84 (0.37)	4.00 (0.00)	0.083 ^†^	3.57 (1.13)	3.57 (0.79)	1.000 ^†^	0.34 (0.25 to 0.43); 0.024
Chair Stand Test (s)	12.23 (3.37)	10.29 (2.39)	0.009	10.66 (1.47)	15.48 (7.83)	0.132	−6.75 (−10.80 to −2.70); 0.002
SPPB Total (Score)	9.84 (1.92)	10.95 (1.03)	0.004	10.86 (1.35)	9.43 (1.27)	0.118	2.53 (1.05 to 4.02); 0.002
GVT 5-m (s)	6.25 (1.63)	5.62 (1.38)	0.036	5.43 (1.09)	6.30 (0.69)	0.020	−1.49 (−2.49 to −0.49); 0.005
GVT Verbal (s)	6.63 (1.71)	6.47 (1.29)	0.657	5.30 (1.10)	7.22 (1.15)	0.017	−2.08 (−3.50 to −0.66); 0.006
GVT Arithmetic (s)	6.82 (1.88)	6.35 (1.51)	0.155	6.26 (1.24)	7.45 (1.52)	0.062	−1.66 (−2.91 to −0.41); 0.012
TUG (s)	12.01 (2.80)	11.03 (3.20)	0.097	11.39 (1.67)	13.37 (1.28)	0.016	−2.96 (−5.02 to −0.89); 0.007
6MWT (m)	414.69 (120.79)	420.11 (101.77)	0.722	425.71 (71.52)	398.50 (65.15)	0.134	29.92 (−26.83 to 86.66); 0.286
Barthel Index (Score)	96.58 (5.79)	98.16 (4.15)	0.084 ^†^	100.00 (0.00)	97.86 (3.93)	0.180 ^†^	0.05 (0.01 to 0.09); 0.044 ^‡^
Muscular performance							
1RM Chest Press (kg)	43.89 (13.77)	52.79 (17.16)	<0.001	34.71 (8.22)	29.33 (5.89)	0.049	12.89 (5.60 to 20.19); 0.001
1RM Leg Press (kg)	100.74 (35.45)	150.63 (47.06)	<0.001	76.43 (25.30)	66.67 (14.46)	0.338	52.73 (38.03 to 67.42); <0.001
Chest Muscle Power (w)	99.00 (48.30)	136.26 (57.56)	<0.001	76.14 (34.58)	52.50 (19.61)	0.022	52.26 (25.67 to 78.86); <0.001
Leg muscle Power (w)	152.26 (86.13)	262.42 (103.92)	<0.001	108.14 (57.21)	72.83 (31.51)	0.071	134.16 (89,91 to 178.40); <0.001 *
Hand Grip (kg)	34.58 (8.35)	34.16 (9.00)	0.618	28.00 (8.71)	28.29 (8.02)	0.811	−0.71 (-3.88 to 2.47); 0.650
Knee Extension (kg)	9.59 (1.76)	11.63 (3.01)	0.013	9.25 (1.76)	8.78 (2.25)	0.701	2.52 (−0.41 to 5.45); 0.089
Flexion Hip (kg)	9.31 (3.23)	10.40 (3.03)	0.044	9.03 (2.98)	7.45 (1.26)	0.244	2.67 (0.39 to 4.94); 0.024
Abduction Hip (kg)	8.05 (2.10)	9.68 (1.86)	0.001	6.55 (1.30)	6.78 (1.83)	0.769	1.39 (−0.24 to 3.02); 0.092

Abbreviations: SD: Standard deviation; SPPB: Short Physical Performance Battery; GVT: Gait Velocity Test; TUG: Timed Up and Go; 6MWT: 6 Minute Walk Test; RM: Repetition maximum. * T test of related samples; + T test of independent samples; ^†^ Wilcoxon; ^‡^ U Mann Whitney. The muscle power output in the propulsive phase was recorded by connecting a velocity transducer to the eGym^®^ machines (T-Force System, Ergotech, Murcia, Spain).

**Table 4:** Results of cognitive functioning outcomes by group.

	Intervention Group (*n* = 19)			Control Group (*n* = 7)			Group Difference ∆ (95% CI) *p*-Value Groups +
	Before	After	*p*-Value *	Before	After	*p*-Value *	
	Mean (SD)	Mean (SD)		Mean (SD)	Mean (SD)		
MMSE (Score)	28.16 (1.07)	28.89 (0.81)	0.005	27.57 (2.82)	27.14 (1.95)	0.407	1.17 (0.19 to 2.14); 0.021
Verbal Fluency Test (Score)	11.37 (5.84)	12.11 (5.36)	0.380	9.43 (5.47)	7.57 (5.38)	0.191	2.59 (−0.61 to 5.80); 0.108
TMT-A (s)	68.32 (38.96)	65.92 (47.34)	0.676	86.10 (62.19)	78.35 (44.36)	0.505	5.34 (−18.17 to 28.86); 0.643

Abbreviations: SD: Standard deviation; MMSE: Mini-mental state examination; TMT-A: Trail making test part A. * T test of related samples; + T test of independent samples.

**Table 5:** Results of health-related quality of life domains by group.

	Intervention Group (*n* = 19)			Control Group (*n* = 7)			Group Difference ∆ (95% CI) *p*-Value Groups +
	Before	After	*p*-Value *	Before	After	*p*-Value *	
	Mean (SD)	Mean (SD)		Mean (SD)	Mean (SD)		
EUROQoL 5D							
EUROQoL 5D ^a^	6.42 (1.35)	5.74 (1.19)	0.038	5.14 (0.38)	6.14 (1.21)	0.038	−1.68 (−2.83 to -0.53); 0.006
EUROQoL 5D Health ^b^	69.05 (12.51)	73.26 (17.53)	0.340	72.29 (21.73)	72.14 (17.76)	0.974	4.35 (−11.28 to 19.99); 0.571
EORTC QLQ-C30 (Functioning scale ^b^)							
Physical	72.20 (26.70)	83.30 (20.90)	0.037	88.60 (15.70)	82.90 (18.00)	0.172 ^†^	16.83 (−0.31 to 33.96); 0.054
Role	88.90 (32.30)	97.20 (11.80)	0.276 ^†^	100.00 (0.00)	100.00 (0.00)	1.000	0.90 (0.84 to 0.96); 0.707 ^‡^
Cognitive	80.60 (25.10)	86.10 (20.00)	0.163	92.90 (18.90)	95.20 (8.10)	0.788 ^†^	3.17 (−13.42 to 19.77); 0.696
Emotional	80.10 (19.80)	82.40 (18.10)	0.686	85.70 (13.40)	85.70 (19.70)	1.000 ^†^	2.31 (−17.91 to 22.54); 0.815
Social	80.60 (25.70)	86.10 (20.80)	0.397 ^†^	100.00 (0.00)	81.00 (27.90)	0.109	0.05 (0.01 to 0.09); 0.062 ^‡^
Global Health status/QoL ^b^	64.80 (21.30)	76.90 (11.60)	0.029 ^†^	77.40 (17.20)	72.60 (14.20)	0.436 ^†^	16.80 (−1.61 to 35.21); 0.072
EORTC QLQ-C30 (Symptom scales and/or items ^c^)							
Fatigue	35.80 (29.40)	30.20 (21.80)	0.464	14.30 (15.30)	14.30 (15.30)	1.000 ^†^	−5.56 (−21.83 to 10.72); 0.485
Nausea and Vomiting	8.30 (24.40)	3.70 (10.80)	0.655 ^†^	0.00 (0.00)	2.40 (6.30)	0.317	0.56 (0.46 to 0.66); 0.362 ^‡^
Pain	32.40 (32.60)	14.80 (21.30)	0.030	14.30 (26.20)	26.20 (30.20)	0.454 ^†^	−29.50 (−60.61 to 1.61); 0.062
Dyspnea	13.00 (20.30)	3.70 (10.80)	0.025 ^†^	14.30 (26.20)	0.00 (0.00)	0.180	0.67 (0.58 to 0.76); 0.817 ^‡^
Sleep Disturbance	29.60 (36.00)	22.20 (34.30)	0.506 ^†^	14.30 (26.20)	14.30 (26.20)	1.000	0.88 (0.82 to 0.94); 0.818 ^‡^
Appetite Loss	25.90 (33.40)	13.00 (28.30)	0.083 ^†^	14.30 (26.20)	19.00 (37.80)	0.655	0.39 (0.29 to 0.49); 0.364 ^‡^
Constipation	22.20 (30.20)	13.00 (23.30)	0.248 ^†^	23.80 (31.70)	33.30 (47.10)	0.577	0.20 (0.12 to 0.28); 0.185 ^‡^
Diarrhea	13.00 (28.30)	18.50 (28.50)	0.603 ^†^	0.00 (0.00)	9.50 (16.30)	0.157	0.92 (0.87 to 0.97); 0.804 ^‡^
Financial Impact	0.00 (0.00)	0.00 (0.00)	1.000 ^†^	4.80 (12.60)	0.00 (0.00)	0.317	0.32 (0.23 to 0.41); 0.109 ^‡^
EORTC QLQ- LC13 ^c^							
Coughing	38.90 (23.60)	24.10 (25.10)	0.104	28.60 (12.60)	23.80 (16.30)	0.356 ^†^	−10.05 (−30.42 to 10.32); 0.318
Hemoptysis	0.00 (0.00)	0.00 (0.00)	1.000 ^†^	4.80 (12.60)	0.00 (0.00)	0.317	0.32 (0.23 to 0.41); 0.109 ^‡^
Dyspnoea	14.20 (15.60)	14.20 (14.70)	1.000	12.70 (20.70)	12.70 (16.30)	1.000 ^†^	0.00 (−11.30 to 11.30); 1.000
Sore Mouth	9.30 (22.30)	14.80 (28.50)	0.546 ^†^	0.00 (0.00)	4.80 (12.60)	0.317	0.98 (0.95 to 1,00); 0.826 ^‡^
Dysphagia	9.30 (22.30)	7.40 (18.30)	0.705 ^†^	0.00 (0.00)	4.80 (12.60)	0.317	0.40 (0.30 to 0.50); 0.225 ^‡^
Peripheral Neuropathy	9.30 (19.20)	13.00 (23.30)	0.414 ^†^	9.50 (25.20)	0.00 (0.00)	0.317	0.18 (0.11 to 0.26); 0.250 ^‡^
Alopecia	5.60 (17.10)	11.10 (22.90)	0.408 ^†^	0.00 (0.00)	19.00 (37.80)	0.180	0.45 (0.35 to 0.55); 0.540 ^‡^
Pain in Chest	13.00 (20.30)	9.30 (15.40)	0.317 ^†^	14.30 (17.80)	4.80 (12.60)	0.157	0.59 (0.49 to 0.69); 0.416 ^‡^
Pain in Arms or Shoulder	13.00 (30.50)	7.40 (21.60)	0.414 ^†^	4.80 (12.60)	0.00 (0.00)	0.317	1.00 (0.97 to 1.00); 0.705 ^‡^
Pain in Other Body Parts	31.50 (35.20)	9.30 (19.20)	0.025 ^†^	14.30 (26.20)	42.90 (46.00)	0.059	0.00 (0.00 to 0.03); 0.007 ^‡^
Categorical Pain Scale ^d^	1.89 (2.35)	1.16 (2.34)	0.317 ^†^	0.57 (1.51)	1.14 (1.95)	0.564 ^†^	0.25 (0.17 to 0.34); 0.216 ^‡^
GDS ^e^	2.79 (1.72)	2.74 (2.66)	0.917	1.57 (1.51)	2.71 (1.80)	0.103	−1.20 (−3.06 to 0.67); 0.197
Borg Scale ^d^	3.67 (0.91)	4.00 (1.33)	0.210	3.14 (0.38)	3.00 (1.67)	1.000	0.33 (−0.88 to 1.55); 0.575

Abbreviations: SD: Standard deviation; EUROQoL 5D: European Quality of Life-5 Dimensions; EORTC: European organization for research and treatment of cancer quality life questionnaire; QoL: quality of life; GDS: Geriatric depression scale; * T test of related samples; + T test of independent samples. ^†^ Wilcoxon; ^‡^ U Mann Whitney. ^a^ The scores range from 5 to 15, with a lower score that represents a better QoL; ^b^ The scores range from 0 to 100, with a higher score representing a higher level of functioning; ^c^ Symptoms and side effects scales and items, scores ranging from 0 to 100, with a higher score representing a higher level of symptoms and side effects; ^d^ The scores range from 0 to 10, the lowest score means no pain or perceived exertion; ^e^ The score shows, normal 0–5, probable depression 6–9, established depression >9 points.

## References

[1] Bray F., Ferlay J., Soerjomataram I., Siegel R.L., Torre L.A., Jemal A. (2018). Global cancer statistics 2018: GLOBOCAN estimates of incidence and mortality worldwide for 36 cancers in 185 countries. CA Cancer J. Clin..

[2] Blandin Knight S., Crosbie P.A., Balata H., Chudziak J., Hussell T., Dive C. (2017). Progress and prospects of early detection in lung cancer. Open Biol..

[3] Hecht S.S., Szabo E. (2014). Fifty Years of Tobacco Carcinogenesis Research: From Mechanisms to Early Detection and Prevention of Lung Cancer. Cancer Prev. Res..

[4] Siegel R., Ward E., Brawley O., Jemal A. (2011). Cancer statistics, 2011: The impact of eliminating socioeconomic and racial disparities on premature cancer deaths. CA Cancer J. Clin..

[5] Baile W.F. (1996). Neuropsychiatric disorders in cancer patients. Curr. Opin. Oncol..

[6] Carlsen K., Jensen A.B., Jacobsen E., Krasnik M., Johansen C. (2005). Psychosocial aspects of lung cancer. Lung Cancer.

[7] Hollen P.J., Gralla R.J., Kris M.G., Potanovich L.M. (1993). Quality of life assessment in individuals with lung cancer: Testing the Lung Cancer Symptom Scale (LCSS). Eur. J. Cancer.

[8] National Comprehensive Cancer Network (NCCN) NCCN Clinical Practice Guidelines in Oncology (NCCN Guidelines^®^) Non-Small Cell Lung Cancer NCCN Evidence BlocksTM Guidelines for patients Non-Small Cell Lung Cancer (Version 1). www.nccn.org/professionals/physician_gls/f_guidelines.asp#nscl.

[9] Sa H., Song P., Ma K., Gao Y., Zhang L., Wang D. (2019). Perioperative Targeted Therapy or Immunotherapy In Non-Small-Cell Lung Cancer. Oncol. Targets Ther..

[10] Michaels C. (2016). The importance of exercise in lung cancer treatment. Transl. Lung Cancer Res..

[11] Peddle-McIntyre C.J., Singh F., Thomas R., Newton R.U., Galvao D.A., Cavalheri V. (2019). Exercise training for advanced lung cancer. Cochrane Database Syst. Rev..

[12] Cavalheri V., Burtin C., Formico V.R., Nonoyama M.L., Jenkins S., Spruit M.A., Hill K. (2019). Exercise training undertaken by people within 12 months of lung resection for non-small cell lung cancer. Cochrane Database Syst. Rev..

[13] Rosero I.D., Ramirez-Velez R., Lucia A., Martinez-Velilla N., Santos-Lozano A., Valenzuela P.L., Morilla I., Izquierdo M. (2019). Systematic Review and Meta-Analysis of Randomized, Controlled Trials on Preoperative Physical Exercise Interventions in Patients with Non-Small-Cell Lung Cancer. Cancers.

[14] Feng S.H., Yang S.T. (2019). The new 8th TNM staging system of lung cancer and its potential imaging interpretation pitfalls and limitations with CT image demonstrations. Diagn. Interv. Radiol..

[15] Reeves B.C., Gaus W. (2004). Guidelines for reporting non-randomised studies. Forsch Komplementarmed Klass Naturheilkd.

[16] Guralnik J.M., Simonsick E.M., Ferrucci L., Glynn R.J., Berkman L.F., Blazer D.G., Scherr P.A., Wallace R.B. (1994). A short physical performance battery assessing lower extremity function: Association with self-reported disability and prediction of mortality and nursing home admission. J. Gerontol..

[17] Brown J.C., Harhay M.O., Harhay M.N. (2015). Physical function as a prognostic biomarker among cancer survivors. Br. J. Cancer.

[18] Campbell K.L., Winters-Stone K.M., Wiskemann J., May A.M., Schwartz A.L., Courneya K.S., Zucker D.S., Matthews C.E., Ligibel J.A., Gerber L.H. (2019). Exercise Guidelines for Cancer Survivors: Consensus Statement from International Multidisciplinary Roundtable. Med. Sci. Sports Exerc..

[19] Harada N.D., Chiu V., Stewart A.L. (1999). Mobility-related function in older adults: Assessment with a 6-min walk test. Arch. Phys. Med. Rehabil..

[20] (2002). ATS statement: Guidelines for the six-minute walk test. Am. J. Respir. Crit. Care Med..

[21] Sánchez-Pérez A., López-Roig S., Pérez A.P., Gómez P.P., Pastor M., Pomares M.H. (2015). Validation Study of the Spanish Version of the Disability Assessment for Dementia Scale. Medicine.

[22] Mlinac M.E., Feng M.C. (2016). Assessment of Activities of Daily Living, Self-Care, and Independence. Arch. Clin. Neuropsychol..

[23] Folstein M.F., Folstein S.E., McHugh P.R. (1975). “Mini-mental state”. A practical method for grading the cognitive state of patients for the clinician. J. Psychiatr. Res..

[24] Saez de Asteasu M.L., Martinez-Velilla N., Zambom-Ferraresi F., Casas-Herrero A., Cadore E.L., Galbete A., Izquierdo M. (2019). Assessing the impact of physical exercise on cognitive function in older medical patients during acute hospitalization: Secondary analysis of a randomized trial. PLoS Med..

[25] Llinas-Regla J., Vilalta-Franch J., Lopez-Pousa S., Calvo-Perxas L., Torrents Rodas D., Garre-Olmo J. (2017). The Trail Making Test. Assessment.

[26] Pickard A.S., Wilke C.T., Lin H.W., Lloyd A. (2007). Health utilities using the EQ-5D in studies of cancer. Pharmacoeconomics.

[27] Bergman B., Aaronson N.K., Ahmedzai S., Kaasa S., Sullivan M. (1994). The EORTC QLQ-LC13: A modular supplement to the EORTC Core Quality of Life Questionnaire (QLQ-C30) for use in lung cancer clinical trials. EORTC Study Group on Quality of Life. Eur. J. Cancer.

[28] González-Barón M., Lacasta M.A., Ordóñez A. (2006). Valoración Clínica en el Paciente con Cáncer.

[29] Brown L.M., Schinka J.A. (2005). Development and initial validation of a 15-item informant version of the Geriatric Depression Scale. Int. J. Geriatr. Psychiatry.

[30] Kurlowicz L., Greenberg S. (2007). The Geriatric Depression Scale (GDS). AJN Am. J. Nurs..

[31] Burkhalter N. (1996). Evaluación de la escala Borg de esfuerzo percibido aplicada a la rehabilitación cardiaca. Rev. Lat.-Am. Enfermagem.

[32] Izquierdo M. (2019). Multicomponent physical exercise program: Vivifrail. Nutr. Hosp..

[33] Guralnik J.M., Ferrucci L., Simonsick E.M., Salive M.E., Wallace R.B. (1995). Lower-extremity function in persons over the age of 70 years as a predictor of subsequent disability. N. Engl. J. Med..

[34] Stuart M., Benvenuti F., Macko R., Taviani A., Segenni L., Mayer F., Sorkin J.D., Stanhope S.J., Macellari V., Weinrich M. (2009). Community-based adaptive physical activity program for chronic stroke: Feasibility, safety, and efficacy of the Empoli model. Neurorehabil. Neural Repair.

[35] Dhillon H.M., Bell M.L., van der Ploeg H.P., Turner J.D., Kabourakis M., Spencer L., Lewis C., Hui R., Blinman P., Clarke S.J. (2017). Impact of physical activity on fatigue and quality of life in people with advanced lung cancer: A randomized controlled trial. Ann. Oncol..

[36] Komaki R., Meyers C.A., Shin D.M., Garden A.S., Byrne K., Nickens J.A., Cox J.D. (1995). Evaluation of cognitive function in patients with limited small cell lung cancer prior to and shortly following prophylactic cranial irradiation. Int. J. Radiat. Oncol. Biol. Phys..

[37] Simo M., Root J.C., Vaquero L., Ripolles P., Jove J., Ahles T., Navarro A., Cardenal F., Bruna J., Rodriguez-Fornells A. (2015). Cognitive and brain structural changes in a lung cancer population. J. Thorac. Oncol..

[38] Chodzko-Zajko W.J., Schuler P., Solomon J., Heinl B., Ellis N.R. (1992). The influence of physical fitness on automatic and effortful memory changes in aging. Int. J. Aging Hum. Dev..

[39] Hill R.D., Storandt M., Malley M. (1993). The impact of long-term exercise training on psychological function in older adults. J. Gerontol..

[40] Dominguez-Sanchez M.A., Bustos-Cruz R.H., Velasco-Orjuela G.P., Quintero A.P., Tordecilla-Sanders A., Correa-Bautista J.E., Triana-Reina H.R., Garcia-Hermoso A., Gonzalez-Ruiz K., Pena-Guzman C.A. (2018). Acute Effects of High Intensity, Resistance, or Combined Protocol on the Increase of Level of Neurotrophic Factors in Physically Inactive Overweight Adults: The BrainFit Study. Front. Physiol..

[41] Stubbs B., Vancampfort D., Hallgren M., Firth J., Veronese N., Solmi M., Brand S., Cordes J., Malchow B., Gerber M. (2018). EPA guidance on physical activity as a treatment for severe mental illness: A meta-review of the evidence and Position Statement from the European Psychiatric Association (EPA), supported by the International Organization of Physical Therapists in Mental Health (IOPTMH). Eur. Psychiatry.

[42] Sweegers M.G., Altenburg T.M., Chinapaw M.J., Kalter J., Verdonck-de Leeuw I.M., Courneya K.S., Newton R.U., Aaronson N.K., Jacobsen P.B., Brug J. (2018). Which exercise prescriptions improve quality of life and physical function in patients with cancer during and following treatment? A systematic review and meta-analysis of randomised controlled trials. Br. J. Sports Med..

[43] Buffart L.M., Kalter J., Sweegers M.G., Courneya K.S., Newton R.U., Aaronson N.K., Jacobsen P.B., May A.M., Galvao D.A., Chinapaw M.J. (2017). Effects and moderators of exercise on quality of life and physical function in patients with cancer: An individual patient data meta-analysis of 34 RCTs. Cancer Treat. Rev..

[44] Rogers L.Q., Courneya K.S., Carter S.J., Anton P.M., Verhulst S., Vicari S.K., Robbs R.S., McAuley E. (2016). Effects of a multicomponent physical activity behavior change intervention on breast cancer survivor health status outcomes in a randomized controlled trial. Breast Cancer Res. Treat..

[45] Patsou E.D., Alexias G.D., Anagnostopoulos F.G., Karamouzis M.V. (2017). Effects of physical activity on depressive symptoms during breast cancer survivorship: A meta-analysis of randomised control trials. ESMO Open.

[46] Chen H.M., Tsai C.M., Wu Y.C., Lin K.C., Lin C.C. (2015). Randomised controlled trial on the effectiveness of home-based walking exercise on anxiety, depression and cancer-related symptoms in patients with lung cancer. Br. J. Cancer.

[47] Meneses-Echávez J.F., González-Jiménez E., Ramírez-Vélez R. (2015). Supervised exercise reduces cancer-related fatigue: A systematic review. J. Physiother..

[48] Vardy J.L., Bell M., Ploeg H.V.D., Turner J., Kabourakis M., Spencer L., Lewis C.R., Hui R., Blinman P.L., Clarke S.J. (2015). The impact of physical activity on fatigue and quality of life in lung cancer patients: A randomised controlled trial (RCT). J. Clin. Oncol..

[49] Oldervoll L.M., Loge J.H., Lydersen S., Paltiel H., Asp M.B., Nygaard U.V., Oredalen E., Frantzen T.L., Lesteberg I., Amundsen L. (2011). Physical exercise for cancer patients with advanced disease: A randomized controlled trial. Oncologist.

[50] Hwang C.L., Yu C.J., Shih J.Y., Yang P.C., Wu Y.T. (2012). Effects of exercise training on exercise capacity in patients with non-small cell lung cancer receiving targeted therapy. Support Care Cancer.

[51] Salhi B., Haenebalcke C., Perez-Bogerd S., Nguyen M.D., Ninane V., Malfait T.L., Vermaelen K.Y., Surmont V.F., Van Maele G., Colman R. (2015). Rehabilitation in patients with radically treated respiratory cancer: A randomised controlled trial comparing two training modalities. Lung Cancer.

[52] Quist M., Rorth M., Langer S., Jones L.W., Laursen J.H., Pappot H., Christensen K.B., Adamsen L. (2012). Safety and feasibility of a combined exercise intervention for inoperable lung cancer patients undergoing chemotherapy: A pilot study. Lung Cancer.

